# The central-peripheral coupling effect of ocular acupuncture kinesitherapy in post-stroke dyskinesia: A functional neuroimaging and neurotic electrophysiology study protocol

**DOI:** 10.3389/fneur.2022.977112

**Published:** 2022-09-01

**Authors:** Di Zhang, Yongshen Wang, Hongpeng Li, Jiang Ma, Jianfeng Sun, Zhipeng Wu, Guilong Zhang, Song Jin

**Affiliations:** ^1^Department of Rehabilitation, Hospital of Chengdu University of Traditional Chinese Medicine, Chengdu, China; ^2^School of Health Preservation and Rehabilitation, Chengdu University of Traditional Chinese Medicine, Chengdu, China; ^3^School of Medicine and Life Science, Chengdu University of Traditional Chinese Medicine, Chengdu, China; ^4^Medical Rehabilitation Department, Affiliated Sport Hospital of Chengdu Sport University, Chengdu, China; ^5^Department of Orthopedics, Hospital of Chengdu University of Traditional Chinese Medicine, Chengdu, China

**Keywords:** ocular acupuncture kinesitherapy, dyskinesia after stroke, fMRI, TMS-MEP, central-peripheral coupling effect

## Abstract

**Background:**

Dyskinesia is a common manifestation after stroke. Motor functional rehabilitation after stroke is of great significance to the maintenance of national health. Ocular Acupuncture Kinesitherapy (OAKT) can repair nerve injuries, improve motor function, reduce rehabilitation time, and promote dyskinesia recovery after stroke. The mechanism, however, remains a mystery, necessitating urgent research. The M1-thalamus-spinal cord neural signaling pathway is linked to limb motor function. Bold-fMRI can represent the cerebral functional state, and TMS-MEP is of certain practical utility for assessing motor neural function and prognosis. Combining fMRI scanning with TMS-MEP detection is predicted to advance brain-spinal cord regulation and muscle response linkage control mechanism research, as well as completely investigate the central-peripheral coupling effect of Ocular Acupuncture Kinesitherapy on dyskinesia after stroke (PSD).

**Methods:**

This is a prospective functional neuroimaging and neurotic electrophysiological study with a case-control design between the PSD with the HC groups and a randomized controlled design within the 3 PSD groups (OAKT group, ocular acupuncture group, and kinesitherapy group). Using fMRI scans and TMS-MEP approach, we will assess the central-peripheral neural function alterations in PSD as well as the coupling effects of OAKT on PSD. We plan to enroll 90 participants at the Hospital of Chengdu University of Traditional Chinese Medicine from Aug 31, 2022, to Dec 31, 2023, including 45 PSD and 45 HC subjects. After enrollment and on the last day after 4-weeks of waiting (HC subjects) or intervention (PSD subjects), all eligible subjects will be evaluated using fMRI scanning, TMS-MEP detection, and the MMT and Fugl-Mayer scales assessment. The MMT and Fugl-Meyer scores will be recorded, and a Pearson correlation analysis will be performed to assess the correlation between clinical and imaging outcomes.

**Discussion:**

Findings of this study will help to explain the central-peripheral coupling effect of OAKT on PSD and to further provide the neural processing of acupuncture kinesitherapy covering the entire pathway from peripheral to central nervous system.

**Clinical trial registration:**

This study is registered with an identifier (ChiCTR2200060483) at the Chinese Clinical Trial Registry in June 2022. http://www.chictr.org.cn/index.aspx.

## Introduction

Dyskinesia after stroke (PSD), a typical result of cerebrovascular accident, is characterized by abnormal muscle strength and tension in the unilateral upper and lower limbs, affecting the patient's ability to sit, stand, walk, and other motor actions. With more than 13 million new cases per year, stroke has become the second leading cause of death and disability worldwide, with high morbidity, mortality, and disability rate. Half of the surviving patients suffer from physical dysfunction to varying degrees, such as motor dysfunction, sensory dysfunction, balance disorder, and so on (1, 2), posing a substantial social and economic burden as well as a significant health risk to humans (3–5). According to one study, PSD has the most recommended recommendations for stroke rehabilitation, with 257 suggestions in six dimensions (6). Actively fostering rehabilitation of post-stroke dyskinesia has an important role in restoring healthy life, enhancing utilization efficiency and logical allocation of medical resources, and lowering the economic burden of patients, families, and even society as a whole (7).

Acupuncture paired with exercise training is a type of integrated Chinese and western therapy that has been shown to dramatically repair nerve injury after stroke, alleviate affected limb functioning, and improve patients' quality of life. Acupuncture has been generally recognized by medical science for its certain success in the treatment of stroke (8, 9), particularly the consciousness and resuscitation restoring needling method, which has been widely used for stroke patients (10–12). Acupuncture with varied techniques based on the level of muscular tension has been found (13) to considerably alleviate the neurological deficit after stroke, and acupuncture has been considered the principal TCM therapy for post-stroke dyskinesia rehabilitation. Exercise training stresses the use of a combination of passive and active exercises to significantly improve neural activity, vascular endothelial function, aerobic metabolism capacity, exercise tolerance, and cardiopulmonary function (14). Muscle strength, proprioception, range of motion, joint stability, and limb motion stimulation can all be improved with exercise training (15). It has become a core means of rehabilitation therapy for a variety of illnesses affecting the skeletal muscle system, endocrine system, cardio-cerebrovascular system, and nervous system. Traditional acupuncture followed by exercise training can improve motor function for PSD more effectively than single acupuncture or exercise training; however, due to the long treatment time, multiple manipulated meridians and acupoints, obvious pain, and complicated steps, it is not conducive to patient long-term persistence and affects the follow-up rehabilitation.

Ocular Acupuncture Kinesitherapy (OAKT) is a form of moving acupuncture in which exercise training is carried out while ocular acupuncture needles are embedded in the orbital tissues. Ocular acupuncture, an acupuncture treatment manipulated on orbit with 8 areas and 13 acupoints, has been approved by the Standardization Administration of China in 2009 (16). As a sort of micro-acupuncture, ocular acupuncture can activate the meridians, invigorate blood, relieve pain and regulate viscera functions by stimulating acupoints around the orbital margin (17–19). Conducting physical therapy (PT), occupational therapy (OT), speech therapy (ST), swallowing training, and other therapies during ocular needle retention can manipulate at fewer points with low pain and simple steps for PSD patients (20), demonstrating the advantages of rapid onset and high patient tolerance. It is advantageous to reduce the treatment length and rehabilitation course, reduce stroke morbidity, and dramatically increase short and long-term efficacy (21–29). In northeast China, OAKT has gradually gained popularity and has become the primary approach for PSD rehabilitation. OAKT has been proven more efficient than traditional acupuncture followed by exercise training in alleviating limb spasms, reducing limb muscle tension, alleviating motor dysfunction, restoring clinical nerve function, and improving daily living ability in PSD (30–36). It has societal significance for its ease of operation, safety and efficiency in treatment, and high patient compliance. The National Administration of Traditional Chinese Medicine established the Operating Standards for OAKT in 2018, clarifying the indications, operating specifications, processes, and precautions in the treatment of stroke and promoting it as a suitable technology for TCM in China. However, because OAKT has such a short application period, it is unclear how it promotes dyskinesia recovery after a stroke.

The brain, as a sophisticated neurological center, can process and integrate input signals by processing and exchanging materials, energy, and information between neurons and synapses to control numerous peripheral organ functions. As a significant input-output channel of motor neuron signal transmission, the Primary Motor Cortex (M1)-Thalamus-Spinal cord is strongly associated with motor functions, such as target-oriented activities of controlling limbs, motor proficiency, coordinated movement, and motor learning (37). The corpus callosum white matter fiber connecting the bilateral primary motor cortex and associated with motor function score was destroyed in post-stroke dyskinesia patients, as was the structural integrity of the right upper longitudinal fasciculus. According to the findings, the cerebral cortex dysfunction of PSD is related to the damaged structure, whereas the contralateral cortex and other brain regions compensate (38), and left upper limb activity and self-paced finger motoring can activate the injured corpus callosum white matter fiber connecting the bilateral primary motor cortex (39).

Functional magnetic resonance imaging (fMRI), a non-interventional functional neuroimaging technique with simultaneous imaging of structure and function, high spatial-temporal resolution, and non-radioactivity, is useful for quantifying cerebral neuronal state and providing an objective reflection of brain functional activities (40). Transcranial Magnetic Stimulation-Motor Evoked Potential (TMS-MEP) is a transcranial magnetic stimulation technique that targets motor cells in the cerebral cortex, spinal nerve roots, and peripheral nerves to record compound action potentials on the relevant muscles, reflecting the functional status of the pyramidal tract. It can be utilized to assess limb motor dysfunction and dynamically monitor recovery conditions due to the great penetration, deep location, and less discomfort of magnetic stimulation (41, 42). TMS-MEP concentrates on the assessment of motor neuronal function, whereas fMRI primarily reflects brain anatomy and functional status. The combination of fMRI and TMS-MEP is intended to thoroughly examine brain functional activity, cortical excitability, and motor neural conduction in pursuit of objective evaluation of motor function from central to peripheral nerves.

We intend to perform fMRI scanning and TMS-MEP technique to observe the OAKT in cerebral function, motor neural function, and dyskinesia improvement to determine the potential mechanism of OAKT in the rehabilitation of PSD. Through correlation analysis of functional neuroimaging, neurotic electrophysiological, and clinical data, it will be contributed to investigating the central-peripheral coupling effect of OAKT on PSD and to further clarify the neurological processing of acupuncture kinesitherapy covering the entire pathway from peripheral to central nervous system.

## Methods

### Study setting

**Figure 1:**
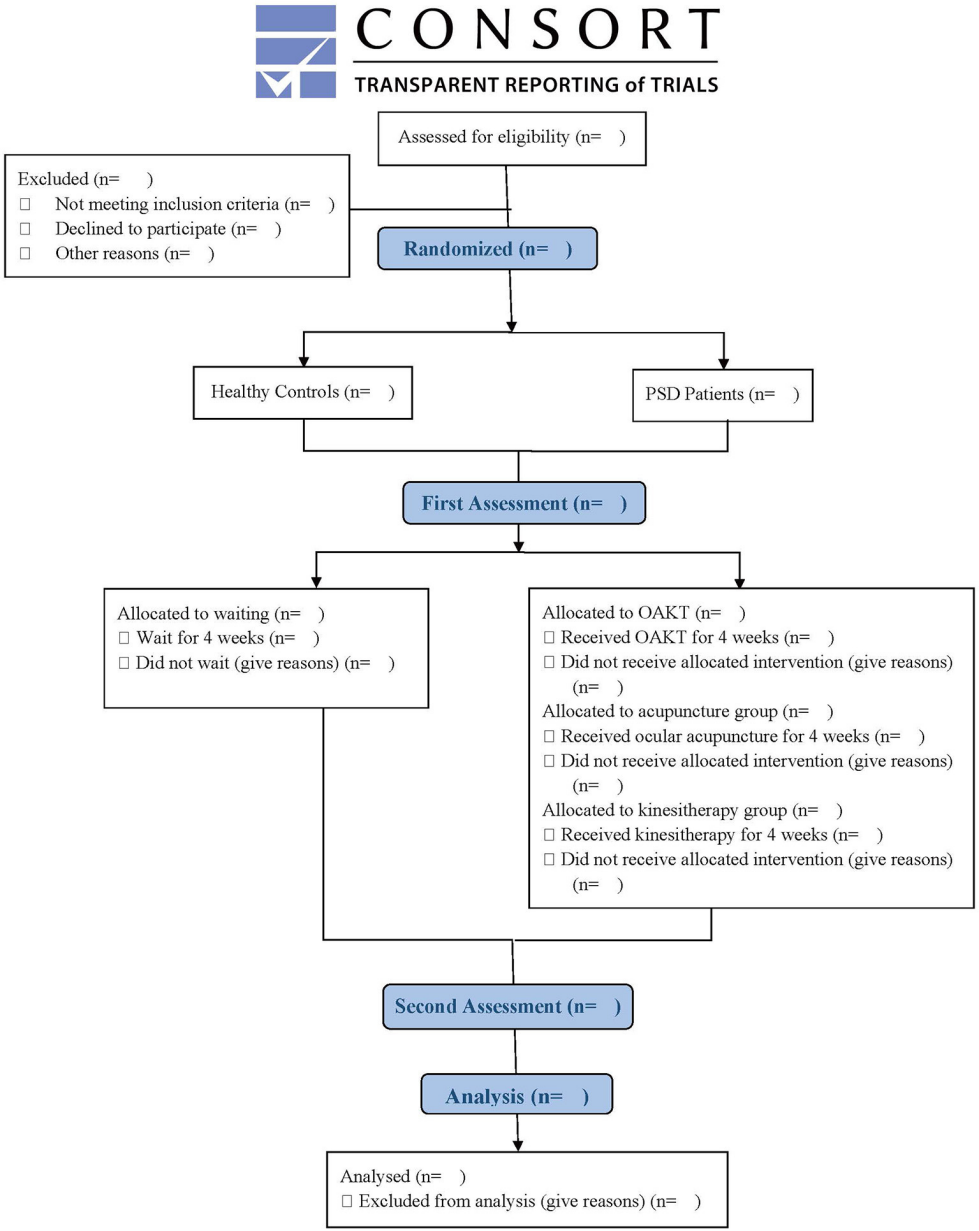
Flow diagram.

This is a prospective functional neuroimaging and neurotic electrophysiological study with a case-control design between the PSD with the HC groups and a randomized controlled design within the 3 PSD groups (OAKT group, ocular acupuncture group, and kinesitherapy group). Using fMRI scanning and the TMS-MEP technique, we will assess the central-peripheral neuronal function changes of PSD as well as the coupling effects of OAKT on them. This trial was authorized by the Institutional Review Board of Chengdu University of Traditional Chinese Medicine in April 2022 (reference number: 2022KL-027) and registered (ChiCTR2200060483) at the Chinese Clinical Trial Registry in June 2022. We plan to enroll 90 participants at the Hospital of Chengdu University of Traditional Chinese Medicine from Aug 31, 2022, to Dec 31, 2023. All eligible subjects will be evaluated by fMRI scanning, TMS-MEP examination, and the MMT and Fugl-Mayer assessment following enrollment and on the last day after 4-weeks of waiting (for HC subjects) or intervention (ocular acupuncture, kinesitherapy, or OAKT for PSD subjects, respectively). MMT and Fugl-Meyer score, ReHo value, cluster size, active or positive brain regions and their MNI coordinates, amplitude (AMP), potential latency (PL), and central motor conduction time (CMCT) in the cortical region will be measured, and Pearson correlation analysis will be performed to assess the relationship between clinical and imaging outcomes. This trial will be conducted in accordance with the Standard Protocol Items: Recommendations for Interventional Trials (SPIRIT) (43). Figure 1 displays a flow diagram.

### Subjects

Patients with post-stroke dyskinesia (PSD) and healthy controls (HC) from the hospital of Chengdu University of Traditional Chinese Medicine and social recruitment, respectively, served as the study's subjects. The diagnostic criteria for patients in this study are based on the Chinese Guidelines for Diagnosis and Treatment of Acute Intracerebral Hemorrhage 2019 (44) and the Chinese Guidelines for Diagnosis and Treatment of Acute Ischemic Stroke 2018 (45). During the anticipated enrollment period from Aug 31, 2022, to December 31, 2023, patients matching the diagnosis standards and satisfying the inclusion and exclusion criteria will be recruited for the study. Table 1 lists the stroke diagnostic criteria.

**Table 1:** Diagnostic criteria of stroke.

**Ischemic stroke**	1. Acute onset
	2. Usually focal or sometimes complete neurologic impairment (unilateral facial or body weakness or numbness, language barriers, and so on)
	3. Imaging lesions or symptoms/signs lasting more than 24 h
	4. Exclude non-vascular etiology
	5. Exclude cerebral hemorrhage by brain CT/MRI scanner
**Hemorrhagic stroke**	1. Acute onset
	2. Usually focal or sometimes complete neurologic impairment accompanied with headache, vomiting, elevated blood pressure, and different degrees of consciousness disorders
	3. Brain CT/MRI scanner showed hemorrhagic focus
	4. Exclude non-vascular etiology.

### PSD participants

This study will exclude dyskinesia brought on by progressive supranuclear palsy, hepatolenticular degeneration, essential tremor, multiple system atrophy, chorea, tossing syndrome, myoclonus, dystonia, Tourette syndrome, and other conditions.

PSD participants that meet the following criteria: (1) have unilateral dyskinesia and a fresh stroke diagnosis; (2) are right-handed and aged 30–80; (3) have no fMRI or TMS contraindications, and no metallic objects inside the body; (4) haven't undergone acupuncture or exercise therapy for a disease-related condition in the previous month; (5) comprehend and agree with the relevant research content and volunteer to sign the informed consent form can be included in this study. While those who (1) have serious illnesses like uncontrollable hypertension, arrhythmia, serious coronary heart disease, uncontrolled diabetes complications, epilepsy, serious organ failure, and a second stroke, etc.; (2) have unstable vital signs; (3) are vulnerable to co-infection, bleeding, tumor, pregnant women, and postoperative dysfunction; (4) receive other Traditional Chinese Medicine rehabilitation interventions besides acupuncture during treatment; (5) with linguistic or cognitive impairment; (6) with a history of mental illness or long-term sedative use; (7) with conditions that prevent exercise will be excluded.

### Healthy controls

Healthy controls that satisfy the requirements: (1) have no history of stroke or impaired motor function; (2) are right-handed between the ages of 30 and 80; (3) have no metallic objects in their bodies and no fMRI or TMS contraindications; (4) have not undergone disease-related acupuncture or exercise therapy in the previous month; (5) understand and agree with relevant research content and volunteer to sign the informed consent form can be included in this study.

While those (1) with significant conditions, such as uncontrollable hypertension, arrhythmia, major coronary heart disease, or diabetes complications are not well-controlled, or have epilepsy, serious organ failure, and second attack of stroke, etc.; (2) with unstable vital signs; (3) are susceptible to co-infection, hemorrhage, tumor, pregnant women, postoperative dysfunction patients; (4) get other Traditional Chinese Medicine rehabilitation intervention other than acupuncture during the waiting period; (5) with linguistic or cognitive impairment; (6) with a history of mental illness or long-term sedative use; (7) with conditions that prevent exercise will be excluded.

### Interventions

PSD participants will be randomized to the ocular acupuncture (OA) group, the kinesitherapy (KT) group, or the OAKT group after enrollment.

**Figure 2:**
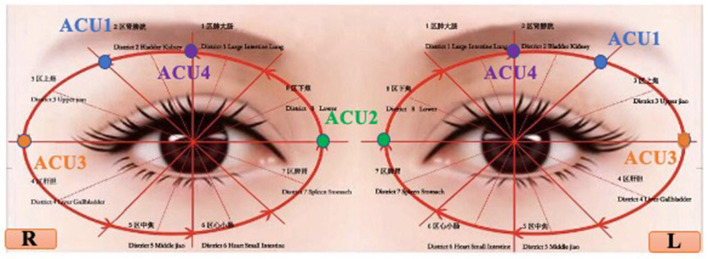
Location of ocular acupoints.

Subjects in the OAKT group will undergo kinesitherapy after receiving ocular acupuncture stimulation at the “Upper-Jiao Area” (ACU 1), “Lower-Jiao Area” (ACU 2), “Liver Area” (ACU 3), and “Kidney Area” (ACU 4) (16) (Figure 2). This involves a series of advised exercise training consisting of warm-up exercises (low levels of aerobic exercise), exercise training (including aerobic training, walking training, treadmill training, resistance training, upper limb flexion adduction-abduction exercise, lower extremity knee flexion-extension exercise, flexibility training), and relaxation training (stretching exercise) (46). Needles for ocular acupuncture will remain embedded until the day's exercise training is finished. OAKT will be manipulated once a day, 5 days a week, for 4 weeks, with 2 days off between courses. Sweating, breathing, pulse, and blood pressure measures will be used to adjust the exercise prescription.

Subjects in the OA group will receive ocular acupuncture stimulation at the same acupoints of that in the OAKT group, without any exercise training of any kind.

Subjects in the KT group will undertake the same kinesitherapy as well as that in the OAKT group, without ocular acupuncture stimulation.

Healthy subjects will only get clinical, functional imaging, and electrophysiological evaluation without any therapy after enrollment and on the last day of the fourth week.

### Outcomes

#### Clinical outcomes

MMT Freehand Muscle Strength Grading and Simplify Fugl-Meyer grading will be evaluated at baseline and study completion. With 11 grades, MMT is used for measuring muscle contraction force based on muscle ability to accomplish gravity-support, anti-gravity, or anti-resistance tasks. Muscle strength increases with grade level. Fugl-Meyer is a motor function evaluation scale that consists of 33 upper-limb and 17 lower-limb subscales. Each subscale is worth 0 to 2 points, for a total score of 100. The motor function improves with a higher score.

#### Functional neuroimaging outcomes

A 3.0 Tesla high-field full-body magnetic resonance imaging system will be used for functional neuroimaging. Bold-fMRI data is acquired in the resting state following structural image scanning with a three-dimensional fast-spoiled gradient-recalled sequence and functional image scanning with a Gradient-Echo-Planar Imaging for 10 min, covering the entire brain, cerebellum, and brain stem. The parameters for scanning structural images include layer a thickness of 1 mm, a Repetition Time/Echo Time (TR/TE) of 2,700 ms/3.39 ms, Field of Vision (FOV) of 256 mm^2^, Matrix of 256 × 256, a Flip Angle of 7°, and an in-plane resolution of 1 × 1 mm. The settings for functional image scanning include a slice thickness of 5 mm, a TR/TE of 2,000 ms/30 ms, Matrix of 64 x 64, FOV of 240 mm^2^, and a Flip Angle of 90°.

The original fMRI data in DICOM format will be copied to a computer and converted by MRIcro software into an analyzable.img file format. The software packages Data Processing Assistant for Resting-State fMRI (DPARSF) and the statistical parametric mapping software package (SPM12) in Matlab (Mathworks, Inc., Natick, Massachusetts) will be utilized to preprocess the fMRI data. Preprocessing steps include (1) realign, all images of each selected subject whose translation in all directions (x, y, z) is <1 mm and rotation angle is <1 degree will be aligned with its first image for further analysis; (2) Spatial normalization, registers images of all subjects to the standard template of Montreal Neurological Institute (MNI) neurological spatial coordinates and re-cuts the voxel to 3 mm 3 for data standardization and individual comparability; (3) slice-timing correction, uses a mathematical approach to collect all images at the same time point by default, with the intermediate time point as the reference layer; (4) smoothing with an 8 mm full width at half maximum (FWHM) kernel to reduce spatial noise, as well as errors caused by spatial normalization; (5) Detrending with linear regression and filtering with a frequency window of 0.01–0.08 Hz to remove low frequency noise caused by respiration, heartbeat, and other physiological factors, as well as high frequency noise from the MRI scanner itself.

Regional homogeneity (ReHo), a typical fMRI analysis, can indirectly reflect the time synchronization of spontaneous neural function activities in a specific brain region. The Kendall's Coefficient Concordance (KCC) will be used to quantify the local consistency of time series between each voxel in the entire brain and the nearest 26 voxels around it by using REST software in Matlab. Each individual receives a ReHo image by computing the KCC value of each voxel. After normalization, the average ReHo image of each subject will be derived by dividing the KCC value of each voxel by the average KCC value of the entire brain. The estimated ReHo value corresponds to the KCC value, which runs from 0 to 1. The closer it is to 1, the more consistent this voxel's activity is in time series with its neighboring voxels, reflecting brain local neuronal activity synchronization. The calculation is expressed as follows:


$$\begin{array}{l} \text{ReHo}=\frac{\sum {\left({\text{R}}_{\text{i}}\right)}^{2}-\text{n}{\left(\text{R}\right)}^{2}}{{\text{k}}^{2}\left({\text{n}}^{3}-\text{n}\right)/12} \end{array}$$

The SPM12 in Matlab will be used to analyze the obtained ReHo value. MNI coordinates of the associated regions, the size of the clusters, and the active or positive brain regions will all be noted before and after the intervention.

#### Neurotic electrophysiology outcomes

Transcranial magnetic stimulation of motor evoked potential (TMS-MEP) examination will be used for neurotic electrophysiology. Cortical magnetic stimulation will be performed using the Keypoint evoked potential instrument and the magnetic stimulator MagII PR030 with standard circular coil and single puls form. The subjects assume a calm supine or seated position. The compound action potentials of the hand muscle (abductor pollicis brevis) will be recorded using a magnetic stimulator center at 175 px on both sides in the C3 and C4 areas, as well as a spinal cord stimulation center in the C7 area. Simultaneously, the compound action potential of the lower extremity muscle (tibialis anterior) will be recorded, with the coil center situated between 2 and 150 px in front of the CZ region. The intensity of the stimulus should gradually increase from low to 65–75% of the maximum output intensity on the scalp and 65–80% of the maximum output intensity on the lower limbs. Varying limbs can be stimulated at different positions and angles depending on the subject's condition. In order to establish the strong repeatability of compound muscle action potential (CMAP) waves, the complete inspection should measure 4–5 CMAP waves. The highest amplitude, shortest latency, and clearest waveform should be recorded.

The cortical region's amplitude (AMP), potential latency (PL), and central motor conduction time (CMCT) serve as the main markers of TMS-MEP. The outcomes can be categorized into 4 levels: (1) no MEP wave with strong repeatability and a clear and stable waveform is evoked during cortical stimulation; (2) prolonged PL in cortical stimulation; (3) prolongation of CMCT; (4) AMP in the affected side drops by more than half when compared to the healthy side. The last three are categorized as prolonged in this study and will be treated as aberrant MEP.

#### Safety outcomes

The incidence of adverse events, primarily fainting, bent needle, and subcutaneous hematoma during ocular acupuncture, tinnitus, chest tightness, palpitation produced by fMRI scanning noise, and muscle soreness or weariness induced by excessive exercise training, will be recorded for safety evaluation.

### Participant timeline

**Figure 3:**
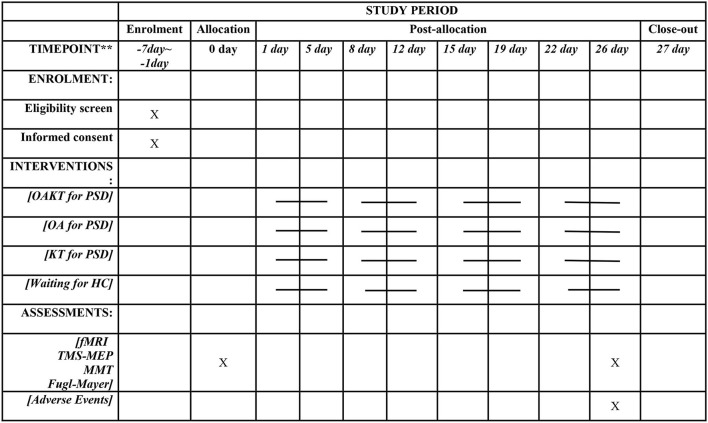
Time schematic diagram of enrolment, interventions, and assessments.

All participants are scheduled to finish the trial by December 2023 once recruitment and data collecting start in Aug 2022. Enrollment, interventions, and participant assessments are depicted in a temporal schematic diagram (Figure 3).

### Sample size

This is a central-peripheral coupling mechanism study rather than a clinical trial. As human-based neuroimaging mechanism studies differ from clinical trials, sample size cannot be estimated using clinical trial computational methods. According to the sample size calculation of imaging studies at home and abroad (47), the sample size is usually 6–12 cases in each group. We determined the sample size as 15 cases per group in accordance with the minimum statistical requirements, considering the drop-out that could be brought on by subjects' compliance and removing the scenario that patients' excessive head movement might influence the data in the statistical analysis progress during fMRI scanning. To keep the statistical results accurate and reliable, 45 healthy controls must be matched simultaneously. In total, 90 subjects-−15 in the OAKT group, 15 in the OA group, 15 in the KT group, and 45 in the HC group—are required for this study.

### Recruitment

All participants are scheduled to finish the trial by December 2023 once recruitment and data collecting start in Aug 2022. Participants who meet the inclusion and exclusion criteria will be recruited after signing an informed consent form prior to the trial. They are free to decide whether or not to take part in the study. Subjects have the option to refuse or withdraw from the study at any time during the procedure. The DMC or researchers may suspend subjects' participation in the study at any time for their good.

### Data collection and management

One researcher (DZ), using a paper version of case report forms (CRF), will gather and document clinical, neurotic electrophysiological, and side-effect information throughout the study. Another researcher (JFS) will copy functional neuroimaging data from the fMRI system using prescribed CDs and record it in an electronic Microsoft Office Excel form with passwords. All data will subsequently be captured and managed using the Research Electronic Data Capture (REDCap) system (48). A researcher (YSW) will enter the data into the REDCap Database, which will then be double-checked by a second researcher (HPL). To ensure accurate data collection and proper data export for further analyses, the statistician (ZPW) will review the database. The Hospital of Chengdu University of Traditional Chinese Medicine will store all information in paper form for 10 years and back it up on various network drives.

### Statistical methods

Only subjects with complete data after 4 weeks of intervention or waiting and pre-post evaluation will be statistically analyzed.

#### Clinical and electrophysiological data analysis

SPSS 23.0 will be used for data analysis. For measurement data, the *T*-test or ANOVA with 95% confidence intervals will be used to compare groups if the distribution is normal, else a Non-parametric Test will be performed. For enumeration data, a chi-square test will be used. A two-sided threshold of *P* < 0.05 will be considered statistically significant.

#### Neuroimaging data analysis

SPM12 software will be used to perform paired *t*-tests on the acquired ReHo value. The statistical threshold voxel-level will be *P* < 0.001 with multiple comparisons corrected by FEW (family-wise error), cluster level α < 0.05. More than 10 continuous voxels will be significant.

#### Correlation analysis between clinical and imaging data

The MATLAB software will be used to conduct the correlation analysis between ReHo values and clinical indexes of PSD. Gender, age, and course will be included as covariates in a Pearson correlation study between the MMT score, Fugl-Meyer score, and ReHo values of PSD. The statistical threshold of *P* < 0.05 will be considered statistically significant.

### Data monitoring

Adverse events include fainting, bent needles, and subcutaneous hematomas during ocular acupuncture, tinnitus, chest tightness, palpitation induced by fMRI scanning noise, and muscle pain and weariness caused by excessive exercise training.

An independent data monitoring committee (DMC) comprised of a study statistician, external stroke researcher, and external rehabilitation therapist will oversee the study and review adverse events. The DMC will constantly monitor the entire study procedure and make suggestions for quality assurance, as well as consider and recommend either stopping or continuing the study if adverse events occur. Once the study or data analysis is finished, the study team will be unblinded.

### Auditing

Once a year, independent staff from the Chengdu University of Traditional Chinese Medicine will monitor and audit the prudent use of project money.

## Discussion

A major side effect of the cerebrovascular accident is PSD, which impairs motor function and places a heavy financial, psychological, and physical burden on patients, families, and society. Rehabilitation after a stroke is crucial to maintaining overall health. Sun (49) verified the superior effects of acupuncture combined with exercise training on neurological function and movement problems in patients with cerebral infarction compared to exercise training alone by measuring Fugl-Meyer score, BI score, Berg score, and NISS score changes (*P* < 0.05). The combination therapy can play a better role in terms of effectiveness and symptom function improvement, with fewer adverse events and good safety, compared to acupuncture or Bobath therapy alone, according to Liu's systematic review of spastic paralysis after stroke (50). The greatest improvement in motor function can be achieved when acupuncture is used in conjunction with exercise training. However, this approach necessitates acupuncture manipulation first, followed by training, which hinders patient long-term perseverance and has an impact on follow-up rehabilitation due to the lengthy treatment time, numerous manipulated meridians and acupoints, obvious pain sensations, and complex steps. By combining ocular acupuncture and exercise training simultaneously, OAKT can ease limb spasms, decrease muscle tension, alleviate motor dysfunction, etc. in PSD patients with improved outcomes. It can minimize the treatment duration and rehabilitation course, and improve short- and long-term efficacy by making use of the advantages of fewer stimulation acupoints, low pain feelings, simple manipulation steps, rapid onset, and high patient tolerance. Unfortunately, due to the short application period, it is yet unclear how OAKT promotes PSD recovery, necessitating urgent research in this area. Therefore, the first scientific challenge of this work is how to appropriately conduct the OAKT mechanism research on PSD.

Through the exchange and fusion of matter, energy, and information, the brain, the higher neurological center, can regulate various functional activities of peripheral organs. The M1-THA-SPC, a crucial input-output pathway of motor neuron signal transmission, can assist in limb goal-oriented motor control, achieving motor proficiency, coordinating movement, and motor learning through the mapping of neurons. Muscle serves as both the peripheral target organ and the primary executive body of the movement in this signaling pathway, the function cannot be carried out without the command and regulation of the higher and secondary centers (brain and spinal cord) (SPC). As a result, we intend to conduct a central-peripheral linkage control study in conjunction with brain-spinal cord regulation and muscular response to understand the mechanism of OAKT on PSD.

fMRI, a non-invasive neuroimaging technique for studying cerebral function, can quantitatively detect the functional activity of brain neurons to reflect the functional state of the brain objectively. Previous fMRI studies have shown that the function of motor-related brain areas, particularly the primary motor cortex, as well as the degree of linkage between the thalamus and other cortical motor networks, is aberrant in stroke patients, and changed dramatically after acupuncture intervention. TMS-MEP is a type of neurotic electrophysiological detection method for recording compound action potentials on the appropriate muscles by transcranial magnetic stimulation of cortical motor cells, spinal nerve roots, and peripheral nerves. It can objectively reflect the current state of cerebral cortex function and is useful in assessing subjects' motor neural function and prognosis. For these reasons, this study will combine fMRI scanning with TMS-MEP detection to realize brain-spinal cord regulation and muscle response linkage control mechanism research, as well as completely investigate the central-peripheral coupling effect of OAKT on PSD.

Two advances in this study are the idea and the methodology. For the idea, we propose to conduct a prospective functional neuroimaging and neurotic electrophysiological study based on a case-control study in the PSD and HC groups as well as a self-control study in the PSD group. The primary motor cortex (higher center), the thalamus (higher center), the spinal cord (secondary center), and the muscle (peripheral) motor neural signal transmission channel are the targets of our assessment of PSD's abnormal central-peripheral neural function and the coupling effects of OAKT on PSD. For the technique, we propose doing a curative effect evaluation and mechanism investigation based on fMRI scanning, TMS-MEP examination, and the MMT and Fugl-Mayer scale assessment to examine the effects of OAKT on brain function, motor nerve function, and degree of motor dysfunction in PSD. Pearson analysis will also be performed between the MMT score, Fugl-Meyer score, and ReHo values of PSD to investigate the association of imaging, neurotic electrophysiological, and clinical outcomes, and to finally exploit the central-peripheral linkage control mechanism and coupling effect of OAKT on PSD from functional imaging and neurotic electrophysiology perspectives.

## Confidentiality

The Hospital of Chengdu University of Traditional Chinese Medicine will maintain complete custody of all participant medical documents (study records/CRF, laboratory sheets, data CDs, etc.). Researchers, ethical committees, and statisticians will have access to medical records. Personal identity will not be disclosed in any public report on the results of this study. We will do everything within the law to protect the privacy of participants' personal medical information.

## Ancillary and post-trial care

The study team will bear all testing and treatment costs for this study.

Any potential harm from this study will be prevented and treated as best we can. The DMC will decide whether a study-related adverse event is caused by the trial's protocol or by the treatment if it occurs. In accordance with the Chinese Quality Management Standards for Drug Clinical Trials, the sponsor will pay for medical expenses and other financial compensation for any harm caused by the testing procedure.

## Ethics statement

The studies involving human participants were reviewed and approved by the Institutional Review Board of Chengdu University of Traditional Chinese Medicine has granted ethics approval for this study, which will be carried out in Aug 2022 in accordance with the Declaration of Helsinki's principles (reference number: 2022KL-027). The patients/participants provided their written informed consent to participate in this study.

## Author contributions

DZ, HL, and YW take responsibility for the concept, design, and integrity of the data. JM, JS, ZW, and GZ: investigation. DZ and YW: methodology. HL: statistical analysis. DZ: project administration, writing–original draft, and funding acquisition. SJ: supervision. GZ: writing–review and editing. All authors contributed to the article and approved the submitted version.

## Funding

This study was supported by the Sichuan Administration of Traditional Chinese Medicine (No. 2020LC0081), the Hospital of Chengdu University of Traditional Chinese Medicine (No. 20ZL02), and the Chengdu University of Traditional Chinese Medicine (No. MPRC2021044).

## Conflict of interest

The authors declare that the research was conducted in the absence of any commercial or financial relationships that could be construed as a potential conflict of interest.

## Publisher's note

All claims expressed in this article are solely those of the authors and do not necessarily represent those of their affiliated organizations, or those of the publisher, the editors and the reviewers. Any product that may be evaluated in this article, or claim that may be made by its manufacturer, is not guaranteed or endorsed by the publisher.

## References

[1] Wu SM Wu B Liu M Chen ZM Wang WZ Anderson CS . Stroke in China: advances and challenges in epidemiology, prevention, and management. Lancet Neurol. (2019) 18:394–405. 10.1016/S1474-4422(18)30500-3 30878104

[2] Li ZY Fang SZ Yu LQ Nie PY Liu F. Meta-analysis of effects of trunk muscle training on lower extremity function in patients with stroke hemiplegia. Fujian Med J. (2020) 42:134–8.

[3] GBD 2016 Dementia Collaborators. Global, regional, and national burden of Alzheimer's disease and other dementias, 1990–2016: A systematic analysis for the global burden of disease study 2016. Lancet Neurol. (2019) 18:88–106. 10.1016/S1474-4422(18)30403-4 30497964 PMC6291454

[4] Lindsay P Norrving B Sacco RL Brainin M Hacke W Martins S . World stroke organization (WSO): global stroke fact sheet 2019. Int J Stroke. (2019) 14:806–17. 10.1177/1747493019881353 31658892

[5] Ekker MS Boot EM Singhal AB Tan KS Debette S Tuladhar AM . Epidemiology, aetiology, and management of ischaemic stroke in young adults. Lancet Neurol. (2018) 17:790–801. 10.1016/S1474-4422(18)30233-3 30129475

[6] Zhang ZX Lv M Luo XF Yu X Lu ZY Wang L . Recommendations of clinical practice guidelines of stroke rehabilitation. Chin J Rehabil Theory Pract. (2020) 26:170–80. 10.3969/j.issn.1006-9771.2020.02.00729490958

[7] World Health Organization Qiu ZY Guo JX Li L. Rehabilitation in the health service system. Chin J Rehabil Theory Pract. (2020) 26:1–14. 10.3969/j.issn.1006-9771.2020.01.003

[8] Yang A Wu HM Tang JL Xu L Yang M Liu G. Acupuncture for stroke rehabilitation. Cochrane Database Syst Rev. (2016) 8:CD004131. 10.1002/14651858.CD004131.pub3 27562656 PMC6464684

[9] Winstein CJ Arena R Bates B Cherney LR Cramer SC Deruyter F . Guidelines for adult stroke rehabilitation and recovery: a guideline for healthcare professionals from the American heart association/American stroke association. Stroke. (2016) 47:e98–l69. 10.1161/STR.0000000000000098 27145936

[10] Shi XM. Clinical study of “Xingnao Kaiqiao” acupuncture on 9005 cases of apoplexy. Guiding J Tradit Chin Med Pharmacol. (2005) 11:3–5. 10.3969/j.issn.1672-951X.2005.01.003

[11] Kou P Shi XM. Review of the curative effect of acupuncture on stroke treated by “Xingnao Kaiqiao” method and analysis of NIHSS scale. Shanxi J Tradit Chin Med. (2015) 13: 41–42+55.

[12] Zhu CT Shi N Shi XM. Clinical study on the intervention time of xing nao kai qiao needling method for hemorrhagic stroke. Shanghai J Acupunct Moxibustion. (2017) 36:1277–80. 10.13460/j.issn.1005-0957.2017.11.1277

[13] Yu XP Yan J Zou W. Clinical observation on the muscle tension staged acupuncture for stroke hemiplegia. Chin Acupunct Moxibustion. (2018) 38:1035–8. 10.13703/j.0255-2930.2018.10.002 30672230

[14] Zhang Q. Literature analysis of TCM intervention in chronic heart failure and clinical effect of exercise rehabilitation. [dissertation]. Shenyang: Liaoning University of Traditional Chinese Medicine (2018). 33371138

[15] Li SC Fu L Guo XC Meng TT Zhang SJ Wang P. Efficacy of exercise therapy on recovery of moto function after anterior cruciate ligament reconstruction: a meta-analysis. Chin J Evid Based Med. (2019) 19:1086–92.

[16] Affiliated Hospital of Liaoning University of Traditional Chinese Medicine Che J Tian WZ Hai Y Tian Y Zhang SW . Standardized Manipulations of Acupuncture and Moxibustion—Part 15: Ophthalmic Acupuncture Techniques. National Public Service Platform for Standards Information. (2021). Available online at: https://std.samr.gov.cn/gb/search/gbDetailedid=D4BEFFF4EA7AB241E05397BE0A0AF581

[17] Wang TD Hai Y. The progress of eye acupuncture relieve pain effect in clinical survey. J Pract Trad Chin Int Med. (2021) 27:182–3. 10.13729/j.issn.1671-7813.2015.07.82

[18] Wang PQ Ju QB Zhou HF Wang J. Study of the theoretical basis of ocular acupuncture therapy based on literature clinical experiment: eye colludes in the brain and regulate viscera. Chin J Basic Med Tradit Chin Med. (2011) 17:1133–4.

[19] Yang T Wang PQ. Study of the specificity of acupoint area based on meridians and anatomy. Hunan J Tradit Chin Med. (2014) 30:99–101.

[20] Li TY Xing HJ Xu YY Shi J Wang JL Sun YH . Features of clinical application of eye acupuncture therapy revealed by data mining. Acupunct Res. (2019) 44:377–82. 31155873 10.13702/j.1000-0607.180495

[21] Tian LR Wang PQ Zhang JM. Analysis of eye acupuncture combined with rehabilitation training for the treatment of stroke. J Emerg Tradit Chin Med. (2021) 30:1970–3. 10.3969/j.issn.1004-745X.2021.11.022

[22] Kong Y. Clinical Study of Eye Acupuncture Combined With Body Acupuncture Therapy in the Treatment of Limb Motor Dysfunction in Patients With Wind-Yang Disturbance Stroke Hemiplegia. Shenyang: Liaoning University of Traditional Chinese Medicine (2021).

[23] Cui C Wang PQ Shao Y Liu J. Effects of transcranial direct current stimulation combined with eye acupuncture exercise therapy on recovery of limb motor dysfunction after stroke. J Liaon Univ Tradit Chin Med. (2021) 23:123–6. 10.13194/j.issn.1673-842x.2021.05.028

[24] Sun Y Li BL Hu N. Observation on the curative effect of eye acupuncture combined with needle retention rehabilitation training in treating hemiplegic limb dysfunction after stroke. Jilin J Chin Med. (2021) 41:272–4. 10.13463/j.cnki.jlzyy.2021.02.036

[25] Gong B. Observation of the Curative Effect of the Rehabilitation Therapy of Eye Acupuncture Retaining Needle on Patients With Spastic Hemiplegia After Stroke. Shenyang: Liaoning University of Traditional Chinese Medicine (2020).

[26] Wang Y Zhou HF Wang Z. A randomized controlled study of periocular acupuncture “inducing resuscitation and unblocking meridians” technique on limb dyskinesia in the acute phase of ischemic stroke. J Liaon Univ Tradit Chin Med. (2020) 22:72–5.

[27] Yang W. Clinical Study of Curative Effect of Eye Acupuncture With Acupuncture Rehabilitation Training in Treating Spastic Hemiplegia of Lower Limbs After Stroke. Shenyang: Journal of Liaoning University of Traditional Chinese Medicine (2020).

[28] Yang S. Clinical Application and Time-Effect Relationship of Rehabilitation With Eye-Acupuncture in Patients With Spasm Stage of Stroke. Shenyang: Liaoning University of Traditional Chinese Medicine (2020).

[29] Zou L Bo Q. Clinical observation eye-acupuncture combine with rehabilitation exercise in the treatment of qi deficiency and blood stasis type stroke hemiplegia at the recovery stage. Clin J Tradit Chin Med. (2018) 30:1247–9. 10.16448/j.cjtcm.2018.0381

[30] Yuan Q. Clinical Study of Eye Acupuncture Combined With Rehabilitation Exercise For Spastic Hemiplegia After Stroke. Hefei: Anhui University of Traditional Chinese Medicine (2016).

[31] Liu H. Clinical Study of Eye Acupuncture With Acupuncture Rehabilitation Therapy on Shoulder Hand Syndrome After Stroke. Shenyang: Liaoning University of Traditional Chinese Medicine (2018).

[32] Wang YR. Clinical Effect of Ophthalmic Needle and Needle Rehabilitation on Apoplexy and Law of Bulbar Conjunctiva Microcirculation. Shenyang: Liaoning University of Traditional Chinese Medicine (2019).

[33] Xu H Wang PQ Chen YG. Clinical observation on eye-acupuncture combined with motion functional training in treatment of ischemic stroke. J Liaon Univ Tradit Chin Med. (2014) 16:123–5.

[34] Li H Lv YX. Clinical observation on 28 cases of motor aphasia caused by cerebral infarction treated by eye acupuncture therapy combined with speech rehabilitation. J N Chin Med. (2014) 46:174–80.

[35] Tian YC Zhang SQ. Clinical observation on eye acupuncture combined with exercise therapy for treating cerebral infarction of spastic hemiplegia. Chin Arch Tradit Chin Med. (2013) 31:674–5.

[36] Duan YP. Controlled clinical observation of eye acupuncture therapy combined with recovery training on hemiplegia after stroke clinical. J Pract Tradit Chin Int Med. (2012) 26:70–2.

[37] Castañeda RM Zingg B Matho KS Chen XY Wang QX Foster NN . Cellular anatomy of the mouse primary motor cortex. Nature. (2021) 598:159–66. 10.1038/s41586-021-03970-w 34616071 PMC8494646

[38] Zhang Y Li KS Ning YZ Fu CH Liu HW Han X . Altered structural and functional connectivity between the bilateral primary motor cortex in unilateral subcortical stroke. Medicine. (2016) 95:e4534. 10.1097/MD.0000000000004534 27495109 PMC4979863

[39] Cui FY. fMRI and DTI Imaging of Hemiplegic Limb Movement and Acupuncture at Yanglingquan Point in brain Functional Remodeling. Beijing: Beijing University of Chinese Medicine (2009).

[40] Fang JL Wang XL. International progress of fMRI study on brain acupuncture effect. Chin Imag J Integ Tradit West Med. (2013) 11:197–202. 10.3969/j.issn.1672-0512.2013.02.033

[41] Chen R Cros D Curra A Lazzaro VD Lefaucheur JP Magistris MR . The clinical diagnostic utility of transcranial magnetic stimulation: report of an IFCN committee. Clin Neurophysiol. (2008) 1l9:504–32. 10.1016/j.clinph.2007.10.014 18063409

[42] Kong XZ Wang YH Li YM Wang YY Meng L . Study on motor evoked potential evaluation of brain function after acute cerebral infarction. Mod J Integ Tradit Chin West Med. (2011) 20:2360–1. 10.3969/j.issn.1008-8849.2011.19.009

[43] Chan AW Tetzlaff JM Gøtzsche PC Altman DG Mann H Berlin JA . SPIRIT 2013 explanation and elaboration: guidance for protocols of clinical trials. BMJ. (2013) 013:1501–7. 10.1136/bmj.e7586 23303884 PMC3541470

[44] Chinese Society of Neurology, Chinese Stroke Society. Chinese guidelines for diagnosis and treatment of acute intracerebral hemorrhage 2019. Chin J Neurol. (2019) 12:994–1005. 10.3760/cma.j.issn.1006-7876.2019.12.003

[45] Chinese Society of Neurology, Chinese Stroke Society. Chinese guidelines for diagnosis and treatment of acute ischemic stroke 2018. Chin J Neurol. (2018) 9:666–82. 10.3760/cma.j.issn.1006-7876.2018.09.004

[46] Chen JY Chen YD Han YL. Expert consensus of exercise rehabilitation after percutaneous coronary intervention. Chin J Interv Cardiol. (2016) 24:361–9. 34475964

[47] Desmond JE Gary HG. Estimating sample size in functional MRI (fMRI) neuroimaging studies: statistical power analyses. J Neurosci Methods. (2002) 118:115–28. 10.1016/S0165-0270(02)00121-8 12204303

[48] Harris PA Taylor R Thielke R Payne J Gonzalez N Conde JG. Research electronic data capture (REDCap)—a metadata-driven methodology and workflow process for providing translational research informatics support. J Biomed Inform. (2009) 42:377–81. 10.1016/j.jbi.2008.08.010 18929686 PMC2700030

[49] Sun S. Effect of acupuncture combined with rehabilitation training on neurological function and dyskinesia in patients with cerebral infarction. Chin Med Mod Dist Educ China. (2020) 18:111–3. 10.3969/j.issn.1672-2779.2020.13.044

[50] Liu DY Ren JW. Acupuncture combined with bobath therapy in treating post-stroke spastic paralysis: A systematic review and meta-analysis. J Guangzhou Univ Tradit Chin Med. (2019) 36:1967–74. 10.13359/j.cnki.gzxbtcm.2019.12.021

